# Reactivation of Multipotency in the Mammary Gland – a Ripple in the Pond and a Turn of the Tide

**DOI:** 10.1007/s10911-025-09586-4

**Published:** 2025-07-17

**Authors:** C. Hager, C. Jehanno, M. Bentires-Alj

**Affiliations:** Department of Biomedicine, University of Basel, University Hospital Basel, Basel, Switzerland

**Keywords:** Multipotency, Lineage Tracing, Mammary Gland, Breast Cancer, Plasticity

## Abstract

Multipotency refers to the ability of a cell to differentiate into multiple, yet limited as opposed to pluripotency, number of cell types within a specific lineage or tissue. Studies using transgenic mouse models of the mammary gland have revealed a cellular hierarchy in which both luminal and basal lineages are replenished by unipotent progenitor cells. Hence, despite the existence of bipotent stem cells, normal mammary gland homeostasis is intimately linked with unipotency. However, recent literature revealed that under specific physiological or experimental conditions, lineage-restricted mammary cells can reacquire multipotency and undergo a lineage switch, challenging the traditional unidirectional model of cell differentiation. This reactivation of multipotency has been observed*,* for instance, in response to pregnancy, lineage ablation or oncogenic stimuli*,* indicating a certain level of plasticity that may have consequences in the context of tumorigenesis. Understanding the molecular mechanisms governing this phenomenon could provide valuable insights into mammary gland cellular hierarchy and breast cancer progression. Indeed, reactivation of multipotency is a result of developed cell plasticity, which can drive tumor heterogeneity, promote disease aggressiveness and hamper diagnosis. This review provides an overview of models that have inferred reactivation of multipotency, discusses the underlying molecular and cellular mechanisms and proposes future perspectives for research.

## Conceptual Introduction

In the 50’s, the biochemical nature of genes had just been discovered by Watson, Crick, Franklin and Wilkins [1–3], therefore arose the question of genesis: How do genes, which are shared by almost every single cell of a living entity, create the physiological heterogeneity of a complex organism? The era of developmental biology began. Among the theories that emerged to conceptually explain this process, the concept of “epigenetic landscape” postulated by Waddington in his book “The strategy of the Genes”, published in 1957, stood the test of time. He envisioned a cell that would travel downhill and differentiate along rugged valleys held by a network of strings representing genes. Depending on the position in the landscape, a cell can lie in a different state of differentiation. This prescient view of cellular differentiation, in which the state of a given cell in space and time depends on the developmental paths of a less differentiated one from which it originates, still holds true today. In the context of mammary gland development, this theoretical view has been elegantly validated by seminal studies that uncovered a switch from multi- to unipotency in progenitor cells during embryonic development [4, 5]. This unidirectional model of cellular differentiation has been challenged in the case of diseases such as cancer, where specific genetic alterations can reprogram lineage-committed cells in a way that they can reacquire multipotency [6–9]. This phenomenon is known as tumor cell plasticity [10] and is a major driver of tumor heterogeneity, which can challenge diagnosis and prognosis, promote therapy resistance and enhance the metastatic potential of disseminated tumor cells [11]. This review will give an overview of the cellular and molecular mechanisms enabling reactivation of multipotency in the mouse mammary gland in physiological, regenerative and oncogenic contexts. Given the differences between the human and murine mammary epithelial hierarchy, the human relevance of these findings will be discussed and put into perspective.

## Methodologies to Study the Adult Mouse Mammary Epithelial Hierarchy

At the cellular level, the mammary gland epithelium (both in mouse and human) is composed of two major cell lineages: a luminal layer comprising luminal progenitors and differentiated hormone sensing cells, and a basal layer comprising mammary stem cells (MaSCs) and myoepithelial cells with a contractile activity enabling mechanical expulsion of milk. Together, these cells form a network of branching ducts which ends in terminal duct lobular units (TDLUs) [12]. Multiple methodologies have been used to gain insights into the cellular hierarchy within the mammary epithelium, ranging from in situ lineage tracing and ex vivo colony passaging to cell transplantation assays, single-cell RNA sequencing (scRNA-seq), and epigenomic profiling of FACS-sorted populations (e.g., ATAC-seq, histone mark ChIP-seq). Each of these techniques possesses strengths and limitations.

Since the experiments by Deome showing that transplanted whole mammary epithelial fragments regenerate an entire epithelial tree-like structure [13], cell transplantation into a de-epithelialized fat pad has been widely used to assess stemness and the capacity to regrow an epithelial structure comprising both luminal and basal lineages [14, 15]. Transplantation of distinct FACS-sorted cells revealed the existence of cells belonging to the basal compartment (defined by the CD29^high^, CD24^low^ profile) that are capable of reforming a complete ductal epithelial tree [14, 16, 17], although the majority of these basal cells failed to reform an epithelial arborescence. This observation sheds light on the heterogeneity of this cell population. While luminal cells failed to reconstitute an epithelial structure when transplanted alone into a cleared fat pad, it has been shown that, when transplanted together, both basal cells and luminal cells contribute to the regeneration of an epithelial tree while preserving their lineage commitment [18]. Despite the relevance of such transplantation assays, it has been proposed that they are rather reflective of a regenerative potential under stress conditions rather than stemness and multipotent capacity in situ.

In contrast, lineage tracing has remained the gold standard assay to monitor cell fate in vivo [19]. This approach uses tissue-specific promoters controlling the expression of the Cre recombinase and subsequent monitoring of the progeny of a chosen cell-of-origin via fluorescence. It can therefore infer the potency status of a given cell type either under physiological conditions, non-physiological conditions or in the context of tumorigenesis (Table 1) [6, 9, 18]. To address the contribution of distinct cell population to the development and maintenance of the mammary gland, several lineage tracing models driven by different promoters of genes specific to basal and luminal lineages have been developed (e.g., *Lgr5, Krt14, Krt5, Procr, Krt8, Krt18*), and yielded both concordant and dissonant results [20]. Under physiological conditions, the tracing of luminal cells revealed their strict unipotency, as they only give rise to cells of the same lineage [4, 6, 9]. However, for the basal cell population, conflicting observations co-exist, owing to the heterogeneity of this cell population and the models used. On the one hand, tracing studies have revealed that *Lgr5* and *Krt14* expressing basal cells are strictly unipotent at adult age [6, 9, 18], and that the switch from multipotency to unipotency occurs during embryonic development [4, 5]. On the other hand, through clonal cell fate mapping using stochastic multicolor Cre reporter driven by the *Krt5, Krt14* and *Lgr5* gene promoters, bipotent MaSCs have been identified in the postnatal mammary gland as key contributors to tissue homeostasis of the mammary gland and to post-pregnancy tissue remodelling [21, 22]. Potential explanations for such discrepancies have been discussed [21]. This second model, therefore, proposes that while unipotent progenitor cells are predominantly contributing to morphogenesis and homeostasis of the mammary gland after birth, rare multipotent MaSCs can coordinate alveologenesis and long-term ductal maintenance [20]. Furthermore, two additional slow-cycling cell populations enriched in the basal layer and defined by the respective PROCR and DLL1 surface markers, have been demonstrated to be enriched in multipotent MaSCs in situ by lineage tracing and/or transplantation assays [23, 24]. Studies from our laboratory have confirmed the findings on PROCR + cells and also revealed their relevance using human reduction mammoplasties (Salvador et al*.*, submitted).

**Table 1:** Summary of Murine Lineage Tracing Models to Study Reactivation of Multipotency in the Mammary Gland

**Reference**	**Lineage Tracing Model**	**Perturbation**	**Effect of Perturbation**
Lilja et al*.* 2018 [5]	Acta2-CreERT2; Rosa-N1ICD-IRES-nGFP Krt5-CreERT2; Rosa-N1ICD-IRES-nGFP	Ectopic expression of active *Notch1* in basal cells	Switch from basal to luminal ERα- lineage specification
Wuidart et al*.* 2018 [4]	Krt14-rtTA;TetO-Cre;Rosa-ΔNp63-IRES-GFP	Ectopic *Trp63* expression in embryonic multipotent progenitors	Promotion of unipotent basal cell differentiation
Wuidart et al*.* 2018 [4]	Krt8-rtTA;TetO-Cre;Rosa-ΔNp63-IRES-GFP	Ectopic *Trp63* expression in adult luminal cells	Conversion of adult luminal cells into basal cells
Song et al*.* 2019 [25]	Krt8-CreERT2;Rosa-mTmG;*Ctnnb1*^*Δexon3/*+^	Activation of Wnt signaling in luminal cells by expressing constitutively activated form of β-catenin	Promotion of LdBC formation
Song et al*.* 2019 [25]	Krt8-CreERT2; Rosa-mTmG;*Ctnnb1*^*fl/fl*^	Deletion of β-catenin in luminal cells	Inhibition of LdBC formation
Song et al. 2019 [25]	Krt8-CreERT2;Rosa-mTmG;Krt14-Lef1Δn	Inhibition of Wnt signaling in LdBCs by expression of dominant negative form of *Lef1*	Inhibition of LdBC formation
Jiang et al*.* 2024 [26]	Ade-Krt5-Cre *Junb*^*fl/fl*^;*Junc*^*fl/fl*^;mTmG	In vitro deletion of *Junb/Junc* in basal cells by adenoviral delivery of Krt5-Cre to mammary gland-derived organoids	Ablation of collagen 1 and ECM stiffness induced basal cell multipotency program
Centonze et al*.* 2020 [27]	Krt5-CreER;Rosa-tdTomato; Krt8-rtTA;TetO-DTA	Ablation of the luminal lineage	Switch from unipotency to multipotency in the basal lineage
Koren et al*.* 2015 [6] Van Keymeulen, et al. 2015 [9]	Krt8-CreERT2;*PIK3CA*^*H1047R*^;tdTomato Krt8-CreERT2;*Pik3ca*^*H1047R*^;Rosa-YFP	*PIK3CA*^*H1047R*^ mutation in luminal cells	Activation of multipotency in the luminal compartment
Koren et al*.* 2015 [6] Van Keymeulen, et al. 2015 [9]	Lgr5-CreERT2;*PIK3CA*^*H1047R*^;td-Tomato Krt5-CreERT2;*Pik3ca*^*H1047R*^;Rosa-YFP	*PIK3CA*^*H1047R*^ mutation in basal cells	Activation of multipotency in the basal compartment
Tao et al*.* 2017 [28]	Krt8-CreERT2;*Trp53*^*fl/fl*^;Rosa-LSL-YFP	Homozygous loss of *Trp53* in luminal cells	Acquisition of basal-like and claudin-low signature
Christin et al*.* 2020 [29]	Sox9-GFP;C3(1);Tag	Inactivation of TRP53 and RB using antigen SV-40	Upregulation of *Sox9* in luminal cells during basal-like tumor induction from luminal progenitors
Christin et al*.* 2020 [29]	MMTV-iCre;*Sox9*^*fl/fl*^;C3(1);Tag	Deletion of *Sox9* in a mouse model of basal-like breast cancer bearing inactivated TRP53 and RB using antigen SV-40	Inhibition of luminal-to-basal cell reprogramming

Finally, single cell transcriptomics largely contributed to the identification of the different cell types forming the mammary gland and of markers capable of discriminating these different populations [30–32]. In regard to the multipotency versus unipotency debate, single cell profiling of the adult mammary gland from the Wahl and Khaled labs have not revealed a population of mammary epithelial cells (MECs) bearing a mixed-lineage gene profile (although the PROCR + population has been detected) [30, 31]. Conversely, single cell profiling of the adult mammary gland from the Visvader lab unveiled the presence of a mixed-lineage population consisting of 5% of the total basal cell population, corroborating the existence of bipotent stem cells [32]. Additionally, the epigenetic profiling of mouse MECs using ATAC-seq revealed unexpectedly that basal cells manifest open chromatin features nearby genes belonging to both lineages, without being associated with productive luminal gene expression [33]. In contrast, ATAC-seq profiling of MECs revealed that luminal progenitor and mature cells exhibit accessible chromatin at the vicinity of luminal genes only, underlying their unipotent potential [33]. Hence, epigenetic profiling might be more suitable for inferring bipotency than transcriptomic profiling, and suggests that certain MECs display epigenetic features recalling multipotency or may be primed to reactivate multipotency.

**Fig. 1:**
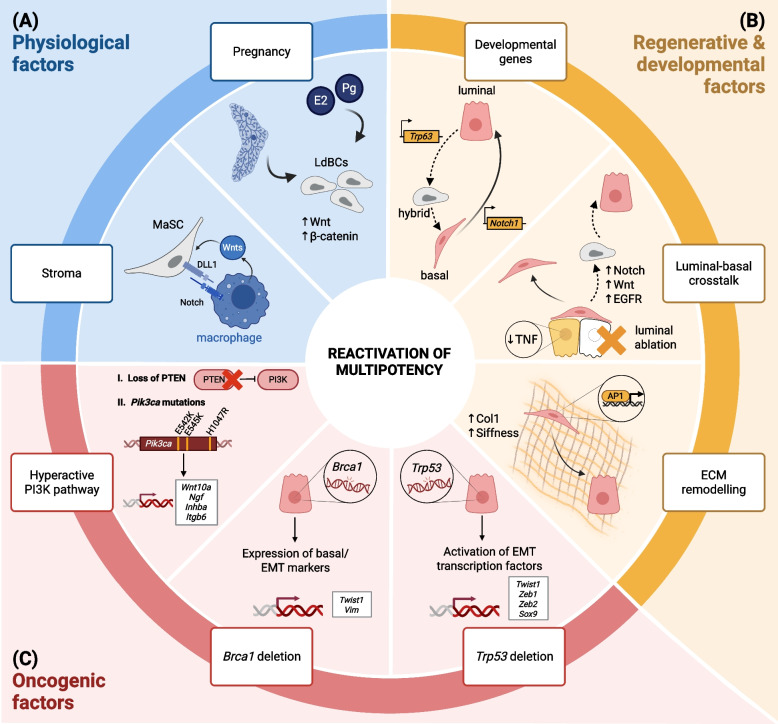
Factors Driving Reactivation of Multipotency in the Mammary Gland Inferred by Lineage Tracing. Reactivation of multipotency in the mammary gland can be achieved by physiological, regenerative/developmental or oncogenic factors. **A** Physiological factors: Pregnancy or hormone supplementation can reactivate multipotency by inducing formation of LdBCs. Stromal cells, such as mammary-resident macrophages, maintain multipotent stem cell activity. **B** Regenerative & developmental factors: Multipotency may be induced by ectopic expression of developmental genes leading to lineage switch. This switch can be gradual as in the case of *Notch1* expression converting basal to luminal cells or via a hybrid intermediate state as in the case of *Trp63* expression, which converts adult luminal to basal cells. Ablation of the luminal lineage reactivates the multipotency of basal cells by abrogating TNF-alpha mediated lineage restriction and by inducing Notch1, Wnt and EGFR signaling in basal cells. Non-cellular factors such as the ECM can reactivate multipotency via increased deposition of collagen 1 and matrix stiffness. **C** Oncogenic factors: Oncogenic hyperactivation of the PI3K pathway or loss of functional *Brca1* or *Trp53* can disrupt lineage commitment. Mutated *PIK3CA* can induce multipotency in either luminal or basal cells, whereas *Brca1* and *Trp53* mutations in luminal cells can activate a basal/EMT gene expression program. Abbreviations: Col1, collagen 1 gene family; ECM, extracellular matrix; EGFR, epidermal growth factor receptor; EMT, epithelial-mesenchymal transition; E2, estrogen; LdBC, luminal-derived basal cell; MaSC, mammary stem cell; Pg, progesterone; TNF, tumor necrosis factor

While powerful, these omics techniques remain largely descriptive. Therefore, functional validation remains essential to prove true multipotency. In the context of this review, we will focus on data showing reactivation of multipotency revealed by lineage tracing in physiological, regenerative and cancer contexts (Table 1, Fig. 1A-C). Prior to that, we must inform our readership that caution is needed when claiming reactivation of multipotency. Indeed, reactivation of multipotency inferred by lineage tracing is considered achieved when fluorescently labelled MECs derived from a unipotent luminal or basal population clonally expand in both lineages. This lineage switch is classically captured by flow cytometry profiling of fluorescent cells with adequate membrane markers that inform about cellular hierarchy. Multipotency can also be functionally demonstrated using the cleared fat-pad transplantation assay. However, the reader must keep in mind that a lineage switch (for instance a luminal-to-basal conversion) does not necessarily imply reactivation of a multipotency program. Indeed, lineage conversion can result from transdifferentiation, which is the process through which a cell transforms into a different cell type without first reverting to a multipotent state. It is experimentally tedious to disentangle the two phenomena. In the context of this review, we will cover reactivation of multipotency inferred by lineage switch and clonal expansion observed under certain experimental conditions, and by transplantation assays. Additionally, the reader should keep in mind that as discrepancies remain regarding the exact potency of *Lgr5* and *Krt14*-expressing basal cells, some interpretations regarding reactivation of multipotency in cells that have been described to be bipotent in other studies may be taken with a grain of salt.

## Regulation of Multipotency at Embryonic and Adult Stages

### Bipotent to Unipotent Switch During Mammary Gland Development

Lineage tracing is an invaluable tool to label and trace cell fate in vivo using lineage-specific promoter activity [34]. Compared to single-color labeling, multicolor clonal analysis offers the advantage of better distinguishing different progenitor-derived clones [5]. Moreover, Cre recombinase inducible systems are key to generating time windows for labeling cells at certain developmental stages and performing chase experiments. Different lineage tracing experiments studying the postnatal mammary gland suggest that progenitor cells contributing to mammary gland maintenance are mostly unipotent after birth, suggesting that lineage restriction occurs during embryonic development. Recent studies have shifted the usage of lineage tracing induction from postnatal to embryonic stages to address the understudied question of how these initially multipotent embryonic MaSCs contribute to postnatal development of the mammary gland [4, 5, 35].

The Confetti reporter system was used for multicolor clonal analysis of luminal and basal lineages [4]. Importantly, lineage tracing postnatally using Krt5-CreERT2;Rosa-Confetti mice demonstrated that *Krt5*-expressing basal cells are unipotent after birth [4]. Additionally, a Notch1-CreERT2 lineage tracing mouse model was used to mark *Notch1*-expressing embryonic multipotent progenitors in the mammary bud [5, 36]. *Notch1* positive mammary cells gave rise to both luminal and basal cells at embryonic stages, but postnatally (i.e., at P3 and onwards) *Notch1*-progeny was exclusively luminal. Similarly, basal cells traced postnatally using the Acta2-CreERT2 model gave rise to basal cells exclusively. This indicates that, at birth, the mammary epithelium is largely restricted to basal and luminal populations that are self-sustained [5].

The pubertal and adult mammary glands are mostly maintained by lineage-committed (i.e., unipotent) stem cells [37]. For instance, at puberty, the luminal lineage can be further divided into two subpopulations, estrogen receptor alpha positive (ERα +) and negative (ERα-), that are maintained by distinct unipotent luminal progenitors [38, 39]. Despite the observation that most epithelial progenitors are unipotent during puberty and adulthood using different lineage tracing models and orthogonal methods such as scRNA-seq and ATAC-seq [18, 40, 41], it remained unclear at what developmental stage lineage restriction occurs and what are the precise mechanisms initiating the switch from multipotency to unipotency [4, 5, 31, 33, 35]. Using Lgr5-GFP lineage tracing models, a FACS-based approach was employed to sort embryonic mammary progenitors (EMPs; CD49f^High^/*Lgr5*-GFP^High^) at E14, adult basal (CD24 +/CD29^High^) and luminal cells (CD24 +/CD29^Low^). Subsequent scRNA-seq on these three populations revealed that EMPs are composed of a homogenous population co-expressing both adult basal and luminal gene sets rather than containing pre-committed basal and luminal subpopulations, corroborating the notion of a unipotency switch. They observed from E14 onwards early signs of lineage segregation marked by increasingly asymmetric expression of luminal (KRT8, SOX9) and basal markers (KRT14, TRP63) [4]. Remarkably, Lilja et al*.* used the Notch1-CreERT2;Confetti mouse line combined with immunofluorescence staining of KRT5 to show the existence of unipotent cells in the murine embryonic mammary bud at the developmental stage E12.5, thus, demonstrating that lineage restriction is a relatively early event in mammogenesis [5]. Independent scRNA-seq studies show that at E15.5, the mammary gland can be transcriptionally dissected into luminal, basal and hybrid cells, confirming the onset of unipotency prior to birth [35]. Alternative to in vivo lineage tracing, pseudo-temporal scRNA-seq can be a useful means to infer differentiation trajectories of embryonic MECs. Using this method, it was proposed that embryonic basal-like cells might either fully acquire basal fate or give rise to luminal cells via an intermediate hybrid-like state [35].

In line with previous work, it was demonstrated in vivo using Rosa-N1ICD-IRES-nGFP mice that constitutively active NOTCH1 receptor in embryonic mammary cells results in their differentiation to luminal ERα- cells postnatally [5]. Thus, NOTCH1 activation is an important mechanism regulating the switch from multipotency to unipotency during mammary gland development. Another cell-intrinsic factor mediating the switch from multipotency to unipotency is *Trp63* [4]. Wuidart et al*.* opted for a doxycycline-inducible approach to trace undifferentiated embryonic (E13) mammary cells expressing *Trp63* transgene in vivo and characterize their differentiation status after birth. Expression of *Trp63* in embryonic mammary cells led to expansion of the basal population, indicative of unipotent basal differentiation [4].

A deeper mechanistic understanding of lineage commitment will provide insights into how reactivation of a multipotency program promotes epithelial cancers [35, 42]. Identifications of suitable early markers of lineage commitment will enable the design of new lineage tracing models and further propel investigation of early mammary progenitor cell fate [35].

### Pregnancy Reactivates Multipotency

Reactivation of multipotency under adult homeostatic conditions, i.e., without exogenous perturbation, has so far only been observed during pregnancy or tumorigenesis (see Sect. “Oncogenic Reactivation of Multipotency”). Thus, pregnancy remains the only known factor to promote luminal-to-basal conversion under normal physiological conditions. The work of Song et al*.* described the emergence of bipotent luminal cells upon pregnancy that are distinct from previously described pregnancy-induced stem cells or parity-identified mammary epithelial cells [25, 43–45]. Luminal cell fate was traced using the Krt8-CreERT2;Rosa-mTmG mouse model, followed by FACS analysis of basal and luminal markers post pregnancy. The authors made the intriguing observation that during early pregnancy, a low percentage of basal cells emerged from the initially GFP + labeled luminal population [25]. The formation of these luminal-derived basal cells (LdBCs) was also achieved by estrogen and progesterone supplementation. Unlike luminal cells that lack the ability to reconstitute a normal mammary gland, both normal basal cells and LdBCs were able to generate a new mammary gland in transplantation experiments [25]. Transcriptomic analyses comparing LdBCs to normal luminal and basal cells strongly suggest that LdBCs are in a hybrid state between basal and luminal identity but are more similar to basal cells [25]. In accordance with previous studies showing the importance of Wnt signaling in basal differentiation, the authors demonstrated that LdBCs expressed higher levels of Wnt pathway associated genes compared to luminal cells [46, 47]. They functionally studied Wnt/β-catenin signaling by constitutively expressing active β-catenin in luminal cells of Krt8-CreERT2;Rosa-mTmG;*Ctnnb1*^*Δexon3/*+^ adult mice, which resulted in expansion of the LdBC population (Table 1, Fig. 1A) [25]. This suggests that overactive Wnt pathway contributes to the re-awakening of multipotency in the adult mammary gland. Moreover, they found a unique ERα-expressing basal cell population within the LdBCs [25]. Further investigation of pregnancy-induced LdBCs will be needed to determine which cells within the luminal population are capable of undergoing such luminal to basal-like transition. These findings urge to question the widely accepted notion that luminal cells are strictly unipotent postnatally and that ERα expression is exclusive to the luminal lineage [48, 49].

### Reactivation of Multipotency by Developmental Factors or Under Regenerative Conditions

Multipotency may also be induced by genetic manipulation. In line with its aforementioned effect in undifferentiated embryonic mammary cells, NOTCH1 activation was sufficient to reprogram committed basal cells to luminal ERα- cells. Ectopic expression of active *Notch1* in committed basal cells was achieved in vivo by crossing Rosa-N1ICD-IRES-nGFP with Krt5-Cre or Acta2-Cre mice (Table 1). Since this reprogramming of adult basal cells occurred in a progressive manner, in which first the basal gene signature was lost before adopting luminal identity, NOTCH1 activation better exemplifies a cell fate switch rather than a reactivation of multipotency [5]. Conversely, ectopic expression of *Trp63* reprogrammed adult luminal cells into basal cells [4]. The luminal lineage tracing model Krt8*-*rtTA;TetO-Cre;Rosa-ΔNp63-IRES-GFP, in which *Trp63* transgene was expressed in the KRT8 + luminal cells four weeks after birth was generated [4] (Table 1). As opposed to *Notch1*-induced cell fate reprogramming, *Trp63*-expressing luminal cells did undergo a hybrid multipotent state before differentiating to unipotent basal cells (Fig. 1B) [4, 5].

Another example of epithelial cell-autonomous mechanisms reactivating multipotency involves the luminal-basal cell crosstalk [27]. Previous work using transplantation assays in vivo have demonstrated that, in the absence of luminal cells, adult basal cells alone can overcome lineage restriction and give rise to luminal and basal cells that reconstitute the mammary gland [14, 16, 27, 50]. This basal cell multipotency is not present when basal and luminal cells are transplanted together, indicating that the luminal cell population might be important in maintaining unipotent basal fate [18, 27]. Centonze et al*.* functionally tested this hypothesis in vivo by using the Krt5-CreER;Rosa-tdTomato;Krt8-rtTA;TetO-DTA mouse model to lineage trace basal cells and ablate luminal cells via tamoxifen and doxycycline-inducible systems, respectively (Table 1). Luminal cell killing in vivo and in ex vivo culture reactivated multipotency in the basal lineage, resulting in basal and luminal cells derived from the tdTomato-traced population. In addition to the classically-defined CD29^low^/EPCAM^high^ luminal cell population, luminal cell ablation also resulted in the emergence of CD29^high^/EPCAM^high^ hybrid cells. This intermediate cell population co-expressed luminal and basal genes and was described to be a transient state along a continuum of luminal cell differentiation, in which basal genes were gradually downregulated. Instead of eliminating the luminal lineage, multipotency in basal cells can also be induced by blocking cell-cell communication between the luminal and basal lineages by inhibiting TNF-alpha, which is upregulated in luminal cells under physiological conditions. Contrarily, inhibition of Notch, Wnt and EGFR signaling decreased the proportion of basal-derived luminal cells after luminal cell ablation, suggesting that these pathways are important promoters of basal cell multipotency upon luminal cell ablation [27] (Fig. 1B).

In addition to cell-intrinsic mechanisms of multipotency re-awakening, non-cell autonomous mechanisms have also been identified to be critical in inducing multipotency. Recent work has shown that extracellular matrix (ECM) composition is another mechanism that can regulate multipotency of MECs [26]. Collagen expression was shown to be universally increased among distinctly-induced multipotent basal cells (i.e., derived either by *PIK3CA*-mutation, basal cell transplantation in vivo, luminal cell ablation or embryonic development). Moreover, by culturing mammary gland organoids derived from adult Krt5-CreER;Rosa-tdTomato transgenic mice, it was shown that increasing concentrations of collagen 1 promoted multipotency in mammary basal cells, resulting in an enrichment of Tomato +/KRT8 + basal-derived luminal cells (Table 1). This effect was also dependent on mechanical cues: Immunofluorescence and FACS analysis of KRT8 levels showed that, in the presence of collagen 1, increasing ECM stiffness also promoted luminal differentiation in the lineage-traced basal cell population. To validate that AP1 mediates basal cell multipotency, they genetically ablated Jun expression in vitro using mammary organoids derived from *Junb*^*fl/fl*^;*Junc*^*fl/fl*^;mTmG mice. Despite the presence of collagen 1 and ECM stiffness, the deletion of *Junb* and *Junc* hampered basal-to-luminal differentiation (Table 1, Fig. 1B). The study suggests that these adult basal cells become multipotent via the collagen 1/integrin β1/FAK/AP1 axis by first transitioning to a hybrid state in which basal and luminal markers are expressed before ultimately shutting down basal gene signature and gradually increasing expression of luminal genes [26].

## Oncogenic Reactivation of Multipotency

A key issue in breast cancer biology consists in identifying the cell-of-origin from which the tumor arises and mutations promoting tumor cell plasticity. Over the last 15 years, the discovery that specific breast cancer mutations can break lineage restriction and reprogram normal MECs was an important breakthrough. It not only provided fundamental insights into the hunt of the putative cell-of-origin of a given tumor type, but also greatly helped to understand the origin of tumor heterogeneity [11, 51]. Whole-genome sequencing analysis of hundreds of human breast tumors unmasked the landscape of somatic mutations and provided fundamental insights into functional characterization of driver versus passenger mutations. Comprehensive analyses of the data revealed the prevalence of certain mutations towards a particular subtype [52–56]. Because the combination of mutations together with the identity and the differentiation state of the cell-of-origin shape the tumor development, understanding the contribution of each individual mutation poses a major challenge [57]. Several mouse models of tumor development using tissue specific and conditional expression of oncogenes or deletion of tumor suppressor genes with or without fluorescent tracers provided fruitful insights into delineating their effects on lineage restriction and the resulting tumor heterogeneity. Several examples of frequent mutations and their impact on lineage commitment of MECs are summarized in the following subsections (Table 1, Fig. 1C).

### Oncogenic PI3K Activation

The phosphoinositide 3-kinase (PI3K) pathway, which is hyperactive in more than 70% of breast cancers due to oncogenic activating mutations (e.g., *Pik3ca*^*E545K*^, *Pik3ca*^*E542K*^ and *Pik3ca*^*H1047R*^), amplification of the alpha catalytic subunit, or the genetic loss of tumor suppressors (*Pten*), is a major driver of cell transformation [58–60]. Mutations in the *PIK3CA* gene are found at similar frequencies in pure ductal carcinoma in situ, ductal carcinoma in situ adjacent to invasive ductal carcinoma (IDC), and in IDC, suggesting that *PIK3CA *mutations are key to shaping the tumor identity at the initial steps of tumorigenesis [61].

Different Cre/rtTA-based transgenic mouse models were generated to elucidate the effects of *Pik3ca* oncogenic variants on lineage commitment and breast tumorigenesis [62]. The mouse mammary tumor virus (MMTV) and the whey acidic protein (WAP) promoters are commonly used for lineage tracing within hormone-sensing epithelial cells and alveolar luminal cells during late pregnancy, respectively [63, 64]. Expression of *Pik3ca-*mutations in luminal cells (e.g., using promoters MMTV, WAP and Krt8) mostly evoked adenosquamous mammary carcinomas, which exhibited mixed-lineage features highlighted by the presence of KRT14/KRT8 & KRT18 double-positive cancer cells [6, 65–67]. These results based on marker expression suggest that luminal-to-basal plasticity had been restored upon oncogenic PI3K signaling. Lineage tracing studies using CreERT2 recombinase expressed from KRT8 + luminal progenitor and mature cells confirmed their clonal expansion in both lineages upon oncogenic PI3K signaling and revealed that reactivation of multipotency causes a mixed-lineage tumor phenotype (Table 1) [6, 9]. Clear fat pad transplantation assays revealed that these LdBCs (Tomato +, CD24^low^, Sca1-) are capable of regenerating a complete bi-lineage epithelial tree, underlining their multipotent capacity [6]. Similarly, unipotent *Krt5* and *Lgr5* expressing cells in these studies were also described to form mammary tumors (mostly adenomyoepitheliomas) upon *Pik3ca*^*H1047R*^ expression, composed of both epithelial cell types revealed by lineage tracing (Table 1) [6]. These results demonstrate that oncogenic PI3K can reprogram unipotent lineage-committed cells in both directions at early stages in the process of tumorigenesis (Fig. 1C). RNA-profiling of MECs that have switched from luminal to basal identity and vice versa revealed that the molecular programs underlying these switches vastly depend on the cells at the origin of the transition, with very few genes found commonly regulated during both switches [6, 9]. Interestingly, the expression of *Pik3ca*^*H1047R*^ in basal *Lgr5* + and luminal *Krt8* + cells induced mammary tumors of distinct histotypes on average after 108 and 78 days, respectively, indicating that aggressiveness and latency greatly depend on the identity of the cell-of-origin [6]. Interestingly, multiple oncogenic mutants of the *PIK3CA* gene have been identified and differentially contribute to tumorigenesis rather than being redundant [65, 68]. Whether they are all potent at restoring multipotency remains to be fully elucidated. Finally, MMTV-Cre and WAP-Cre driven conditional deletion of *Pten*, encoding a phosphatase that controls PI3K pathway activation, results in heterogeneous tumor formation consisting primarily of adenomyoepithelioma expressing markers of both epithelial lineages including alpha smooth muscle actin (αSMA), basal and luminal cytokeratins (KRT8, KRT5, KRT14) and ERα, thus, reinforcing that this pathway is key to regulating lineage plasticity [69, 70].

### *Brca1* Deletion

It has long been speculated that basal-like breast cancers originate from a basal cell-of-origin as the resulting tumor lacks steroid hormone receptors and expresses specific markers restricted to the normal basal cells, such as αSMA, KRT5 or KRT14 [71–73]. Transgenic mouse models of *Brca1* loss driven tumors therefore became widely used and confirmed that its genetic ablation within basal cells (using Krt14-Cre in *Trp53*^+/−^ heterozygous background) resulted in tumors of the basal-like subtype [7, 74–76]. However, despite the seemingly logical conclusion of basal cell-of-origin giving rise to basal-like tumors and absence of lineage disruption, the resulting histological adenomyoepithelioma tumor type did not resemble the invasive adenocarcinoma histotype detected in the vast majority of basal-like breast cancers [7]. Additionally, an increased proportion of luminal progenitors has been observed in the human breast of *BRCA1* mutation carriers [77], suggesting that they may be the cell-of-origin of basal-like breast cancer. The generation of a *β*-galactosidase Blg-Cre allele predominantly expressed in luminal progenitors driving *Brca1* deletion (*Trp53*^+/−^ heterozygous background) strikingly lead to the formation of basal-like tumors that phenocopy the human invasive adenocarcinoma phenotype [7]. FACS profiling revealed accumulation of progenitor cells (CD24^low^), typical of basal cells, indicating that *Brca1* loss can overcome lineage restriction. As the authors wisely stated, the Blg-Cre *Brca1*^−/−^ tumors expressed epithelial-mesenchymal transition (EMT)-associated genes such as *Vim* and *Twist1*, which are also a feature of normal myoepithelial cells in the mammary gland (Fig. 1C) [7]. It is consequently not clear to what extent expression of EMT markers in these tumors reflect a case of reactivation of multipotency rather than an event of EMT promoting luminal-to-basal conversion, thus, opening the question of similarities between basal cell fate and EMT programs. The example of *Brca1* therefore suggests that EMT, which is classically associated with the metastatic cascade [78], can also be a major feature of tumor cell heterogeneity by overcoming luminal lineage restriction and shaping tumor identity.

### *Trp53* Deletion

Several publications reported that the TP53 tumor suppressor acts as a barrier to somatic cell reprogramming [79–81], thereby uncovering an effect of TP53 on differentiation processes. In the context of breast cancer, previous studies demonstrated that *Trp53* mutations are frequent in the preneoplastic stages of mouse mammary tumor development and in ductal carcinoma in situ, suggesting their importance in driving tumor development and identity [82, 83]. The influence of *Trp53* deficiency in the luminal lineage has been inquired mostly using the Krt8-CreERT2 model (Table 1) [28]. After 4 weeks of tracing, the homozygous loss of *Trp53* led to clonal expansion of cell islets especially positive for ERα expression, without loss of luminal fate, as YFP + marked cells retained a luminal profile (CD24^high^, CD29^low^). However, homozygous deletion of *Trp53* led to mammary tumor formation with high penetrance (100% of the mice) after 6 months on average. RNA profiling of those tumors, unexpectedly, revealed an inclination towards the basal-like and claudin-low subtype, confirmed by the strong expression of *Krt14* and low expression of various claudin 3, 4 and 7 genes. The elevated expression of EMT transcription factors such as *Twist*, *Zeb1* and *Zeb2* might explain these observations, thus recalling the BRCA1 example [28]. *Sox9* has also been shown to be upregulated following *Trp53* inactivation (Sox9-GFP;C3(1);Tag model), leading to NFκB signaling in and luminal-to-basal conversion of progenitor cells, and basal-like tumorigenesis (Table 1, Fig. 1C) [29]. These observations are consistent with the characterization of mammary tumors that formed in the MMTV-Cre *Trp53*^+/−^ model as poorly differentiated adenocarcinoma or spindle cell/EMT, presenting regions enriched in KRT14 +/KRT8 + cells [84]. Additionally, targeted deletion of both *Rb* and *Trp53* in bipotent stem cells led to histologically uniform, aggressive, EMT-type tumors, while *Rb* deletion alone triggered luminal B tumor formation [85]. Finally, deletion of *Trp53* in the basal compartment using the Krt14-Cre allele resulted in basal-like tumors expressing both basal (KRT5) and luminal (KRT8/18) cytokeratins, clearly indicating lineage defects [75]. These data highlight that *Trp53* deletion predominantly dictates tumor fate towards a poorly differentiated phenotype [85, 86]. Finally, as previously mentioned, numerous publications reporting the influence of BRCA1 deficiency were carried out in a *Trp53*^+/−^ heterozygous background, enforcing the contribution of *Trp53* deletion towards the basal-like subtype [7, 74].

The reactivation of multipotency driven by oncogenic alterations early in the process of tumorigenesis raises several outstanding questions: Is the reactivation of multipotency a prerequisite for initiating tumorigenesis? Are all MECs equally susceptible to undergoing lineage plasticity and reactivation of multipotency? Given the heterogeneity of epithelial cell populations, does the level of luminal or basal differentiation act as a barrier to lineage reprogramming? What are the consequences of oncogenic transformation of bipotent MaSCs, such as PROCR + cells? How do cells that have undergone reactivation of multipotency behave in response to systemic, targeted or immune checkpoint-based anti-cancer therapies? Finally, how do these cells differ in terms of metastatic ability [87]? These basic questions need to be addressed in order to leverage this knowledge into therapeutic actionability.

## Human Relevance

This overview of reactivation of multipotency in the mouse mammary gland, inferred by lineage tracing in different models upon different biological contexts, raises the question of the human relevance of such findings. This question is difficult to address owing to the impossibility of performing in situ lineage tracing using fluorescent tracers, although some tracing experiments using ex vivo models have emerged in the literature in other contexts [88]. The human epithelial hierarchy has been established both via FACS, mammosphere-forming and transplantation assays, as well as single-cell sequencing. It has common and distinct features compared to the mouse mammary gland. Indeed, FACS profiling of human mammary epithelial cells (HMECs) revealed the existence of a similar luminal population consisting of both luminal mature and progenitor cells, as well as a basal cell population comprising both myoepithelial cells and MaSCs, which mostly differ from those of mice in terms of markers. Within the epithelial cell populations, the expression of EPCAM delineates luminal cells with EPCAM +/ALDH +/CD49f + and EPCAM +/ALDH-/CD49f + and CD49f- being enriched in luminal progenitors and luminal mature cells, respectively. The basal compartment is delineated by the EPCAM-/CD10 + profile which comprises distinct clusters of MaSCs (3 in total) and myoepithelial cells [77, 89]. The difficulty of using direct in vivo lineage tracing approaches in humans leaves limited options to study the commitment of a cell to a certain lineage, such as comparative omics approaches or marker expression studies. Despites differences in the mammary epithelial hierarchy between human and murine species, genetically engineered mouse models recapitulate some aspects of molecular human breast tumor heterogeneity but not all. Indeed, while basal-like cancers represent only 15% of the cases in humans, it appears that the majority of tumors resulting from transgenic models exhibit features of this subtype. Tumors arising from the luminal cells are either mixed-lineage upon *Pik3ca* mutations, basal-like upon *Brca1* deletion, basal-like and claudin-low upon *Trp53* mutations or basal-like and normal-like upon *Pten* deletion, underlying lineage switch (see Sect. “Oncogenic Reactivation of Multipotency”). This suggests that the cell-of-origin of luminal cancer in murine models has not yet been clearly defined, and that reactivation of multipotency may be a prerequisite for tumor initiation [51]. It may also indicate that genetic mouse models are not appropriate to study the biology of hormone dependent ERα + and PR + cancer, and that distinct species may have different breast cancer subtype incidences. For example, cats (and felines in general) predominantly develop hormone receptor negative (basal-like or HER2-amplified) mammary tumors that frequently metastasize to different organs, such as the lungs [90–92], in contrast to women, in whom 70% of the diagnosed tumors are hormone receptor-positive. Interestingly, rats, in contrast to mice, seem to be a promising model for studying ERα + cancer, as they develop spontaneous or induced mammary tumors that resemble human hormone-receptor positive breast cancer [93–95].

Little is known on lineage switch and reactivation of multipotency in human breast tumors. Lineage defects in luminal models were detected at late disease stages upon endocrine therapy pressure (giredestrant) in ERα + patient-derived xenograft models, leading to KRT14 expression [96]. For basal-like breast cancer, it has been reported that these tumors are more heterogenous and can express luminal markers such as KRT7, KRT18 or vitamin D receptor [97]. Whether this apparent basal-like tumor heterogeneity reflects lineage disruption at the initial steps of tumorigenesis or the differentiation state of the cell-of-origin remains elusive. In the context of *Brca1* loss [77], both human and murine data support the luminal progenitor origin of basal-like breast cancer. Lineage defects have also been detected in human *BRCA1* mutant specimens by imaging mass cytometry (IMC) [98]. Comprehensive scRNA-seq analyses of human breast tumors revealed expression of EMT genes across different histological subtypes and uncovered changes in stromal and immune cells comprising the tumor microenvironment [99]. These non-cancer cell autonomous changes might be important for the reactivation of multipotency. For instance, cancer-associated fibroblasts in *BRCA*-mutant tumors expressed increased levels of collagen genes, which may lead to changes in the ECM composition that can activate a multipotency program in the tumor cells [26, 99].

In the context of the normal breast, immunofluorescence and marker expression uncovered dissimilarities between human and mouse mammary epithelial hierarchies. In the scientific community, there is a mistaken assumption coming from mouse data that basal cytokeratins are never expressed in the luminal cells in human [100]. Indeed, massive multiplex immunostaining of thousands of normal human mammary epithelial sections demonstrated that the cytokeratins (KRT5/14/17) are also expressed in the luminal layer of normal human breast lobules (as compared to ducts) [97]. This observation questions the paradigm of strict lineage restriction which does not necessarily hold true in the human mammary gland and rather highlights the underestimated diversity among stem and progenitor populations. The exploration of the single-cell profiling data of the human adult breast (> 800 000 cells across 55 donors) also confirmed the existence of cells co-expressing luminal and basal cytokeratins [101]. These cells have been proposed to be “lineage‐primed” and may represent transient intermediates prior to luminal lineage commitment [99]. This observation may be of importance in the context of cancer, as invasive lobular carcinoma (ILC) differs in terms of marker expression and is characterized particularly by the loss of E-Cadherin compared to IDC [102]. Whether lobular cells are more prone to plasticity remains to be elucidated, although it may be experimentally challenging due to the absence of lobules in adult virgin mice, in contrast to humans.

Finally, an extensive evaluation by IMC of the ability of organoid culture technology to preserve stem/progenitor and differentiated cell types was reported (79 different organoid lines, 38 markers). Comparative analysis of different organoids grown from basal/stem, luminal progenitor and mature to their native counterparts revealed that organoids can preserve their epithelial lineages over long-term culture [103]. It therefore appears that organoid models can be used in the future as a versatile tool to address the influence of miscellaneous breast cancer mutations on lineage commitment of HMECs, although the culture conditions seem to be essential. Indeed, the authors also demonstrate that the composition of the organoid medium can affect the mammary epithelial cell lineages [104]. For example, they observed that removal of EGF causes an increase in the relative proportion of mature luminal cells with a concomitant decrease in basal cells, while removal of either Neuregulin, p38 MAPK inhibitor, or FGF7/FGF10 results in a relative decrease in mature luminal cells with a concomitant increase in other mammary lineages.

## Concluding Remarks

Tissue homeostasis in the adult mouse mammary gland is intimately linked to the unipotency of basal and luminal progenitors. Reactivation of multipotency is achieved by perturbation of this homeostasis, for instance, during pregnancy and hormonal changes, under regenerative conditions, or under oncogenic alterations and ECM remodeling. Multipotency is thus far from being only a cell-intrinsic feature; it also depends on the surrounding microenvironment, which dynamically evolves depending on the hormonal status or, for instance, in the context of tumorigenesis [99]. In multiple glandular epithelia (mammary, prostate or salivary gland), lineage commitment results indeed from a paracrine crosstalk between the different epithelial and stromal cell types comprising the gland. It is indeed remarkable that normal luminal and basal lineages ensure mutual fate commitment by heterotypic communication, thus, restraining the activity of multiple signaling pathways controlling potency (Wnt or Notch). In the prostatic epithelium, inflammatory JAK/STAT3 signaling and androgen deprivation have also been linked to induction of plasticity, lineage disruption and re-acquisition of stem properties [105, 106]. In the context of tumorigenesis, reactivation of multipotency by oncogenic perturbation is a major driver of intra-tumoral heterogeneity. It is highly probable that not all cells within the mammary gland are equally susceptible to reactivation of multipotency, which in turn highlights the importance of the cell-of-origin for tumor identity. Consequently, additional work is required to identify cells that are likely to undergo such plasticity and fuel intra-tumor heterogeneity, as well as to elucidate the underlying mechanisms, in order to leverage therapeutic actionability. Also, it has been reported that at the initial steps of the metastatic cascade, disseminated tumor cells exhibit stem-like transcriptional features, and that cell clusters containing KRT14 + cells have a high metastatic ability. These data suggest that reactivation of multipotency, at least transiently, may enhance the metastatic ability of tumor cells [10]. In conclusion, it appears that no matter how robust lineage commitment may seem in a healthy adult mammary gland, it only takes a perturbation of the homeostasis or one genetic hit, like a ripple in the pond, to reveal its fragility and to embark committed cells onto a different lineage trajectory that might ultimately represent a crucial step towards tumor development.

## Data Availability

No datasets were generated or analysed during the current study.

## References

[1] Wilkins MHF, Stokes AR, Wilson HR. Molecular Structure of Nucleic Acids: Molecular Structure of Deoxypentose Nucleic Acids. Nature. 1953. 10.1038/171738a0.13111175

[2] Franklin RE, Gosling RG. Molecular Configuration in Sodium Thymonucleate. Nature. 1953. 10.1038/171740a0. 13054694 10.1038/171740a0

[3] Watson JD, Crick FHC. Molecular Structure of Nucleic Acids: a Structure for Deoxyribose Nucleic Acid. Nature. 1953. 10.1038/171737a0. 13054692 10.1038/171737a0

[4] Wuidart A, et al. Early Lineage Segregation of Multipotent Embryonic Mammary Gland Progenitors. Nat Cell Biol. 2018. 10.1038/s41556-018-0095-2. 29784918 10.1038/s41556-018-0095-2 PMC5985933

[5] Lilja AM, et al. Clonal Analysis of Notch1-expressing Cells Reveals the Existence of Unipotent Stem Cells that Retain Long-term Plasticity in the Embryonic Mammary Gland. Nat Cell Biol. 2018;20:677. 29784917 10.1038/s41556-018-0108-1 PMC6984964

[6] Koren S, et al. PIK3CAH1047R Induces Multipotency and Multi-lineage Mammary Tumours. Nature. 2015. 10.1038/nature14669. 26266975 10.1038/nature14669

[7] Molyneux G, et al. BRCA1 Basal-like Breast Cancers Originate From Luminal Epithelial Progenitors and not From Basal Stem Cells. Cell Stem Cell. 2010. 10.1016/j.stem.2010.07.010. 20804975 10.1016/j.stem.2010.07.010

[8] Hein SM, et al. Luminal Epithelial Cells Within the Mammary Gland can Produce Basal Cells Upon Oncogenic Stress. Oncogene. 2016;35:1461. 26096929 10.1038/onc.2015.206 PMC4688047

[9] Van Keymeulen A, et al. Reactivation of Multipotency by Oncogenic PIK3CA Induces Breast Tumour Heterogeneity. Nature. 2015. 10.1038/nature14665. 26266985 10.1038/nature14665

[10] Jehanno C, Vulin M, Richina V, Richina F. Phenotypic Plasticity During Metastatic Colonization. Trends Cell Biol. 2022:1–14. 10.1016/j.tcb.2022.03.007. 10.1016/j.tcb.2022.03.007 35484037

[11] Koren S, Bentires-Alj M. Breast Tumor Heterogeneity: Source of Fitness, Hurdle for Therapy. Mol Cell. 2015. Preprint at 10.1016/j.molcel.2015.10.031. 26590713 10.1016/j.molcel.2015.10.031

[12] Visvader JE, Stingl J. Mammary Stem Cells and the Differentiation Hierarchy: Current Status and Perspectives. Genes Dev. 2014. Preprint at 10.1101/gad.242511.114. 24888586 10.1101/gad.242511.114 PMC4052761

[13] Deome KB, Faulkin LJ, Bern HA, Blair PB. Development of Mammary Tumors From Hyperplastic Alveolar Nodules Transplanted Into Gland-free Mammary Fat Pads of Female C3H Mice*. http://aacrjournals.org/cancerres/article-pdf/19/5/515/2374297/crs0190050515.pdf. 13663040

[14] Shackleton M, et al. Generation of a Functional Mammary Gland From a Single Stem Cell. Nature. 2006. 10.1038/nature04372. 16397499 10.1038/nature04372

[15] Smith GH, Medina D. Re-evaluation of Mammary Stem Cell Biology Based on in Vivo Transplantation. Breast Cancer Res. 2008;10. Preprint at 10.1186/bcr1856. 10.1186/bcr1856 PMC2374966 18304381

[16] Stingl J, et al. Purification and Unique Properties of Mammary Epithelial Stem Cells. Nature. 2006. 10.1038/nature04496. 16395311 10.1038/nature04496

[17] Sleeman KE, et al. Dissociation of Estrogen Receptor Expression and in Vivo Stem Cell Activity in the Mammary Gland. J Cell Biol. 2007. 10.1083/jcb.200604065. 17190790 10.1083/jcb.200604065 PMC2063618

[18] Van Keymeulen A, et al. Distinct Stem Cells Contribute to Mammary Gland Development and Maintenance. Nature. 2011. 10.1038/nature10573. 21983963 10.1038/nature10573

[19] Van Amerongen R, Bowman AN, Nusse R. Developmental Stage and Time Dictate the Fate of Wnt/β-catenin- Responsive Stem Cells in the Mammary Gland. Cell Stem Cell. 2012. 10.1016/j.stem.2012.05.023. 22863533 10.1016/j.stem.2012.05.023 PMC13155203

[20] Fu NY, Nolan E, Lindeman GJ, Visvader JE. Stem Cells and the Differentiation Hierarchy in Mammary Gland Development. Physiol Rev. 2020;100:489–523. 31539305 10.1152/physrev.00040.2018

[21] Rios AC, Fu NY, Lindeman GJ, Visvader JE. In Situ Identification of Bipotent Stem Cells in the Mammary Gland. Nature. 2014. 10.1038/nature12948. 24463516 10.1038/nature12948

[22] De Visser KE, et al. Developmental Stage-specific Contribution of LGR5+ Cells to Basal and Luminal Epithelial Lineages in the Postnatal Mammary Gland. J Pathol. 2012. 10.1002/path.4096. 22926799 10.1002/path.4096

[23] Chakrabarti R, et al. Notch Ligand Dll1 Mediates Cross-talk Between Mammary Stem Cells and the Macrophageal Niche. Science. 2018. 10.1126/science.aan4153. 29773667 10.1126/science.aan4153 PMC7881440

[24] Wang D, et al. Identification of Multipotent Mammary Stemcells by Protein C Receptor Expression. Nature. 2015. 10.1038/nature13851. 25327250 10.1038/nature13851

[25] Song W, et al. Hormones Induce the Formation of Luminal-derived Basal Cells in the Mammary Gland. Cell Res. 2019. 10.1038/s41422-018-0137-0. 30631153 10.1038/s41422-018-0137-0 PMC6460434

[26] Jiang C, et al. Collagen Signaling and Matrix Stiffness Regulate Multipotency in Glandular Epithelial Stem Cells in Mice. Nature Commun. 2024;15:10482. 39695111 10.1038/s41467-024-54843-5 PMC11655882

[27] Centonze A, et al. Heterotypic Cell–cell Communication Regulates Glandular Stem Cell Multipotency. Nature. 2020;584:608. 32848220 10.1038/s41586-020-2632-y PMC7116172

[28] Tao L, Xiang D, Xie Y, Bronson RT, Li Z. Induced P53 Loss in Mouse Luminal Cells Causes Clonal Expansion and Development of Mammary Tumours. Nat Commun. 2017. 10.1038/ncomms14431. 28194015 10.1038/ncomms14431 PMC5316831

[29] Christin JR, et al. Stem Cell Determinant SOX9 Promotes Lineage Plasticity and Progression in Basal-like Breast Cancer. Cell Rep. 2020;31:107742. 32521267 10.1016/j.celrep.2020.107742 PMC7658810

[30] Bach K, et al. Differentiation Dynamics of Mammary Epithelial Cells Revealed by Single-cell RNA Sequencing. Nat Commun. 2017. 10.1038/s41467-017-02001-5. 29225342 10.1038/s41467-017-02001-5 PMC5723634

[31] Giraddi RR, et al. Single-cell Transcriptomes Distinguish Stem Cell State Changes and Lineage Specification Programs in Early Mammary Gland Development. Cell Rep. 2018. 10.1016/j.celrep.2018.07.025. 30089273 10.1016/j.celrep.2018.07.025 PMC6301014

[32] Pal B, et al. Construction of Developmental Lineage Relationships in the Mouse Mammary Gland by Single-cell RNA Profiling. Nat Commun. 2017. 10.1038/s41467-017-01560-x. 29158510 10.1038/s41467-017-01560-x PMC5696379

[33] Dravis C, et al. Epigenetic and Transcriptomic Profiling of Mammary Gland Development and Tumor Models Disclose Regulators of Cell State Plasticity. Cancer Cell. 2018. 10.1016/j.ccell.2018.08.001. 30174241 10.1016/j.ccell.2018.08.001 PMC6152943

[34] van de Moosdijk AAA, Fu NY, Rios AC, Visvader JE, van Amerongen R. Lineage Tracing of Mammary Stem and Progenitor Cells. Methods Mol Biol. 2017;1501:291. 27796960 10.1007/978-1-4939-6475-8_15

[35] Carabaña C, et al. Spatially Distinct Epithelial and Mesenchymal Cell Subsets Along Progressive Lineage Restriction in the Branching Embryonic Mammary Gland. EMBO J. 2024;43:2308–36. 38760574 10.1038/s44318-024-00115-3 PMC11183262

[36] Rodilla V, et al. Luminal Progenitors Restrict Their Lineage Potential During Mammary Gland Development. PLoS Biol. 2015;13:e1002069. 25688859 10.1371/journal.pbio.1002069 PMC4331521

[37] Davis FM, et al. Single-cell Lineage Tracing in the Mammary Gland Reveals Stochastic Clonal Dispersion of Stem/progenitor Cell Progeny. Nat Commun. 2016;7:13053. 27779190 10.1038/ncomms13053 PMC5093309

[38] Van Keymeulen A, et al. Lineage-restricted Mammary Stem Cells Sustain the Development, Homeostasis, and Regeneration of the Estrogen Receptor Positive Lineage. Cell Rep. 2017. 10.1016/j.celrep.2017.07.066. 28813665 10.1016/j.celrep.2017.07.066 PMC5575359

[39] Wang C, Christin JR, Oktay MH, Guo W. Lineage-biased Stem Cells Maintain Estrogen-receptor-positive and -negative Mouse Mammary Luminal Lineages. Cell Rep. 2017;18:2825. 28329676 10.1016/j.celrep.2017.02.071 PMC5408864

[40] Wuidart A, et al. Quantitative Lineage Tracing Strategies to Resolve Multipotency in Tissue-specific Stem Cells. Genes Dev. 2016. 10.1101/gad.280057.116. 27284162 10.1101/gad.280057.116 PMC4911926

[41] Scheele CLGJ, et al. Identity and Dynamics of Mammary Stem Cells During Branching Morphogenesis. Nature. 2017;542:313. 28135720 10.1038/nature21046 PMC6097610

[42] Gupta PB, Pastushenko I, Skibinski A, Blanpain C, Kuperwasser C. Phenotypic Plasticity: Driver of Cancer Initiation, Progression, and Therapy Resistance. Cell Stem Cell. 2019;24. Preprint at 10.1016/j.stem.2018.11.011. 10.1016/j.stem.2018.11.011 PMC7297507 30554963

[43] Boulanger CA, Wagner KU, Smith GH. Parity-induced Mouse Mammary Epithelial Cells are Pluripotent, Self-renewing and Sensitive to TGF-β1 Expression. Oncogene. 2005;24:552. 15580303 10.1038/sj.onc.1208185

[44] Matulka LA, Triplett AA, Wagner KU. Parity-induced Mammary Epithelial Cells are Multipotent and Express Cell Surface Markers Associated With Stem Cells. Dev Biol. 2007;303:29. 17222404 10.1016/j.ydbio.2006.12.017

[45] Asselin-Labat ML, et al. Control of Mammary Stem Cell Function by Steroid Hormone Signalling. Nature. 2010;465:798. 20383121 10.1038/nature09027

[46] Chakrabarti R, et al. Δnp63 Promotes Stem Cell Activity in Mammary Gland Development and Basal-like Breast Cancer by Enhancing Fzd7 Expression and Wnt Signalling. Nat Cell Biol. 2014;16:1004. 25241036 10.1038/ncb3040 PMC4183725

[47] Lindley LE, et al. The WNT-controlled Transcriptional Regulator LBH is Required for Mammary Stem Cell Expansion and Maintenance of the Basal Lineage. Development. 2015;142:893. 25655704 10.1242/dev.110403 PMC4352974

[48] Sleeman KE, et al. Dissociation of Estrogen Receptor Expression and in Vivo Stem Cell Activity in the Mammary Gland. J Exp Med. 2007. 10.1084/jem2041oia1. 10.1083/jcb.200604065 PMC2063618 17190790

[49] Asselin-Labat ML, et al. Steroid Hormone Receptor Status of Mouse Mammary Stem Cells. J Natl Cancer Inst. 2006;98:1011. 16849684 10.1093/jnci/djj267

[50] Prater MD, et al. Mammary Stem Cells Have Myoepithelial Cell Properties. Nat Cell Biol. 2014;16:942–50. 25173976 10.1038/ncb3025 PMC4183554

[51] Skibinski A, Kuperwasser C. The Origin of Breast Tumor Heterogeneity. Oncogene. 2015;34:5309–16. 25703331 10.1038/onc.2014.475 PMC4734640

[52] Curtis C, et al. The Genomic and Transcriptomic Architecture of 2,000 Breast Tumours Reveals Novel Subgroups. Nature. 2012. 10.1038/nature10983. 22522925 10.1038/nature10983 PMC3440846

[53] Nik-Zainal S, et al. Landscape of Somatic Mutations in 560 Breast Cancer Whole-genome Sequences. Nature. 2016. 10.1038/nature17676. 27135926 10.1038/nature17676 PMC4910866

[54] Stephens PJ, et al. The Landscape of Cancer Genes and Mutational Processes in Breast Cancer. Nature. 2012. 10.1038/nature11017. 22722201 10.1038/nature11017 PMC3428862

[55] Koboldt DC, et al. Comprehensive Molecular Portraits of Human Breast Tumours. Nature. 2012. 10.1038/nature11412. 23000897 10.1038/nature11412 PMC3465532

[56] Pereira B, et al. The Somatic Mutation Profiles of 2,433 Breast Cancers Refines Their Genomic and Transcriptomic Landscapes. Nat Commun. 2016. 10.1038/ncomms11479. 27161491 10.1038/ncomms11479 PMC4866047

[57] Visvader JE. Cells of Origin in Cancer. Nature. 2011. Preprint at 10.1038/nature09781. 21248838 10.1038/nature09781

[58] Fruman DA, et al. The PI3K Pathway in Human Disease. Cell. 2017. Preprint at 10.1016/j.cell.2017.07.029. 28802037 10.1016/j.cell.2017.07.029 PMC5726441

[59] Cantley LC. The Phosphoinositide 3-kinase Pathway. Science. 2002. Preprint at 10.1126/science.296.5573.1655. 12040186 10.1126/science.296.5573.1655

[60] Miller TW, Rexer BN, Garrett JT, Arteaga CL. Mutations in the Phosphatidylinositol 3-kinase Pathway: Role in Tumor Progression and Therapeutic Implications in Breast Cancer. Breast Cancer Res. 2011. Preprint at 10.1186/bcr3039. 22114931 10.1186/bcr3039 PMC3315683

[61] Miron A, et al. PIK3CA Mutations in in Situ and Invasive Breast Carcinomas. Cancer Res. 2010. 10.1158/0008-5472.CAN-08-2660. 20551053 10.1158/0008-5472.CAN-08-2660 PMC2905503

[62] Koren S, Bentires-Alj M. Mouse Models of PIK3CA Mutations: One Mutation Initiates Heterogeneous Mammary Tumors. FEBS J. 2013. Preprint at 10.1111/febs.12175. 23384338 10.1111/febs.12175

[63] Nusse R, Varmus HE. Many Tumors Induced by the Mouse Mammary Tumor Virus Contain a Provirus Integrated in the Same Region of the Host Genome. Cell. 1982;31:99–109. 6297757 10.1016/0092-8674(82)90409-3

[64] Pittius CW, et al. A Milk Protein Gene Promoter Directs the Expression of Human Tissue Plasminogen Activator CDNA to the Mammary Gland in Transgenic Mice. Proc Natl Acad Sci. 1988;85:5874–8. 2842753 10.1073/pnas.85.16.5874 PMC281867

[65] Meyer DS, et al. Expression of PIK3CA Mutant E545K in the Mammary Gland Induces Heterogeneous Tumors but is Less Potent Than Mutant H1047R. Oncogenesis. 2013. 10.1038/oncsis.2013.38. 24080956 10.1038/oncsis.2013.38 PMC3816227

[66] Meyer DS, et al. Luminal Expression of PIK3CA Mutant H1047R in the Mammary Gland Induces Heterogeneous Tumors. Cancer Res. 2011. 10.1158/0008-5472.CAN-10-3827. 21482677 10.1158/0008-5472.CAN-10-3827

[67] Tikoo A, et al. Physiological Levels of Pik3caH1047R Mutation in the Mouse Mammary Gland Results in Ductal Hyperplasia and Formation of ERα-positive Tumors. PLoS One. 2012. 10.1371/journal.pone.0036924. 22666336 10.1371/journal.pone.0036924 PMC3364244

[68] Yu K, et al. PIK3CA Variants Selectively Initiate Brain Hyperactivity During Gliomagenesis. Nature. 2020. 10.1038/s41586-020-1952-2. 31996845 10.1038/s41586-020-1952-2 PMC7577741

[69] Kim KM, et al. Combined Expression of Protein Disulfide Isomerase and Endoplasmic Reticulum Oxidoreductin 1-α is a Poor Prognostic Marker for Non-small Cell Lung Cancer. Oncol Lett. 2018;16:5753–60. 30344729 10.3892/ol.2018.9339 PMC6176373

[70] Li G, et al. Conditional Loss of PTEN Leads to Precocious Development and Neoplasia in the Mammary Gland. Development. 2002. 10.1242/dev.129.17.4159 12163417

[71] Livasy CA, et al. Phenotypic Evaluation of the Basal-like Subtype of Invasive Breast Carcinoma. Mod Pathol. 2006. 10.1038/modpathol.3800528. 16341146 10.1038/modpathol.3800528

[72] Nielsen TO, et al. Immunohistochemical and Clinical Characterization of the Basal-like Subtype of Invasive Breast Carcinoma. Clin Cancer Res. 2004;10:5367–74. 15328174 10.1158/1078-0432.CCR-04-0220

[73] Yehiely F, Moyano JV, Evans JR, Nielsen TO, Cryns VL. Deconstructing the Molecular Portrait of Basal-like Breast Cancer. Trends Mol Med. 2006. Preprint at 10.1016/j.molmed.2006.09.004. 17011236 10.1016/j.molmed.2006.09.004

[74] Liu X, et al. Somatic Loss of BRCA1 and P53 in Mice Induces Mammary Tumors With Features of Human BRCA1-mutated Basal-like Breast Cancer. Proc Natl Acad Sci U S A. 2007. 10.1073/pnas.0702969104. 17626182 10.1073/pnas.0702969104 PMC1924557

[75] Hollern DP, et al. A Mouse Model Featuring Tissue-specific Deletion of P53 and Brca1 Gives Rise to Mammary Tumors With Genomic and Transcriptomic Similarities to Human Basal-like Breast Cancer. Breast Cancer Res Treat. 2019. 10.1007/s10549-018-5061-y. 30484104 10.1007/s10549-018-5061-y PMC6418066

[76] Smart CE, et al. Targeted Disruption of Brca1 in Restricted Compartments of the Mouse Mammary Epithelia. Breast Cancer Res Treat. 2008. 10.1007/s10549-007-9859-2. 18095153 10.1007/s10549-007-9859-2

[77] Lim E, et al. Aberrant Luminal Progenitors as the Candidate Target Population for Basal Tumor Development in BRCA1 Mutation Carriers. Nat Med. 2009. 10.1038/nm.2000. 19648928 10.1038/nm.2000

[78] Chaffer CL, San Juan BP, Lim E, Weinberg RA. EMT, Cell Plasticity and Metastasis. Cancer Metastasis Rev. 2016. 10.1007/s10555-016-9648-7. 27878502 10.1007/s10555-016-9648-7

[79] Kawamura T, et al. Linking the P53 Tumour Suppressor Pathway to Somatic Cell Reprogramming. Nature. 2009. 10.1038/nature08311. 19668186 10.1038/nature08311 PMC2735889

[80] Hong H, et al. Suppression of Induced Pluripotent Stem Cell Generation by the P53–p21 Pathway. Nature. 2009. 10.1038/nature08235. 19668191 10.1038/nature08235 PMC2917235

[81] Marión RM, et al. A P53-mediated DNA Damage Response Limits Reprogramming to Ensure IPS Cell Genomic Integrity. Nature. 2009. 10.1038/nature08287. 19668189 10.1038/nature08287 PMC3624089

[82] Done SJ, Arneson NCR, Özçelik H, Redston M, Andrulis IL. P53 Mutations in Mammary Ductal Carcinoma in Situ But not in Epithelial Hyperplasias. Cancer Res. 1998. 9485035

[83] Joseph Jerry D, et al. Mutations in P53 are Frequent in the Preneoplastic Stage of Mouse Mammary Tumor Development. Cancer Res. 1993. 8324748

[84] Adams JR, et al. Cooperation Between Pik3ca and P53 Mutations in Mouse Mammary Tumor Formation. Cancer Res. 2011. 10.1158/0008-5472.CAN-10-0738. 21324922 10.1158/0008-5472.CAN-10-0738

[85] Jiang Z, et al. Rb Deletion in Mouse Mammary Progenitors Induces Luminal-B or Basal-like/EMT Tumor Subtypes Depending on P53 Status. J Clin Investig. 2010. 10.1172/JCI41490. 20679727 10.1172/JCI41490 PMC2929714

[86] Knight JF, et al. Met Synergizes With P53 Loss to Induce Mammary Tumors that Possess Features of Claudin-low Breast Cancer. Proc Natl Acad Sci U S A. 2013. 10.1073/pnas.1210353110. 23509284 10.1073/pnas.1210353110 PMC3619286

[87] Cheung KJ, Gabrielson E, Werb Z, Ewald AJ. Collective Invasion in Breast Cancer Requires a Conserved Basal Epithelial Program. Cell. 2013;155:1639. 24332913 10.1016/j.cell.2013.11.029 PMC3941206

[88] Bury LAD, Fu S, Wynshaw-Boris A. Neuronal Lineage Tracing From Progenitors in Human Cortical Organoids Reveals Novel Mechanisms of Human Neuronal Production, Diversity, and Disease. bioRxiv. 2023. 10.1016/j.celrep.2024.114862 39395167

[89] Morel AP, et al. A Stemness-related ZEB1-MSRB3 Axis Governs Cellular Pliancy and Breast Cancer Genome Stability. Nat Med. 2017. 10.1038/nm.4323. 28394329 10.1038/nm.4323

[90] Caliari D, et al. Triple-negative Vimentin-positive Heterogeneous Feline Mammary Carcinomas as a Potential Comparative Model for Breast Cancer. BMC Vet Res. 2014. 10.1186/s12917-014-0185-8. 25249140 10.1186/s12917-014-0185-8 PMC4180584

[91] Cannon CM. Cats, Cancer and Comparative Oncology. Vet Sci. 2015. Preprint at 10.3390/vetsci2030111. 29061935 10.3390/vetsci2030111 PMC5644631

[92] Beha G, et al. Molecular Phenotype of Primary Mammary Tumours and Distant Metastases in Female Dogs and Cats. J Comp Pathol. 2014. 10.1016/j.jcpa.2013.07.011. 24060151 10.1016/j.jcpa.2013.07.011

[93] Nicotra R, Lutz C, Messal HA, Jonkers J. Rat Models of Hormone Receptor-positive Breast Cancer. J Mammary Gland Biol Neoplasia. 2024;29. Preprint at 10.1007/s10911-024-09566-0. 10.1007/s10911-024-09566-0 PMC11196369 38913216

[94] Gil Del Alcazar CR, et al. Insights Into Immune Escape During Tumor Evolution and Response to Immunotherapy Using a Rat Model of Breast Cancer. Cancer Immunol Res. 2022;10:680. 35446942 10.1158/2326-6066.CIR-21-0804 PMC9177779

[95] Bu W, Li Y. Advances in Immunocompetent Mouse and Rat Models. Cold Spring Harb Perspect Med. 2024;14:a041328. 37217281 10.1101/cshperspect.a041328 PMC10810718

[96] Liang J, et al. ERα Dysfunction Caused by ESR1 Mutations and Therapeutic Pressure Promotes Lineage Plasticity in ER+ Breast Cancer. Nat Cancer. 2025. 10.1038/s43018-024-00898-8. 39805955 10.1038/s43018-024-00898-8

[97] Santagata S, et al. Taxonomy of Breast Cancer Based on Normal Cell Phenotype Predicts Outcome. J Clin Investig. 2014. 10.1172/JCI70941. 24463450 10.1172/JCI70941 PMC3904619

[98] Gray GK, et al. A Human Breast Atlas Integrating Single-cell Proteomics and Transcriptomics. Dev Cell. 2022;57:1400. 35617956 10.1016/j.devcel.2022.05.003 PMC9202341

[99] Pal B, et al. A Single-cell RNA Expression Atlas of Normal, Preneoplastic and Tumorigenic States in the Human Breast. EMBO J. 2021;40:e107333. 33950524 10.15252/embj.2020107333 PMC8167363

[100] Dontu G, Ince TA. Of Mice and Women: a Comparative Tissue Biology Perspective of Breast Stem Cells and Differentiation. J Mammary Gland Biol Neoplasia. 2015. Preprint at 10.1007/s10911-015-9341-4. 26286174 10.1007/s10911-015-9341-4 PMC4595531

[101] Reed AD, et al. A Single-cell Atlas Enables Mapping of Homeostatic Cellular Shifts in the Adult Human Breast. Nat Genet. 2024;56:652. 38548988 10.1038/s41588-024-01688-9 PMC11018528

[102] Ciriello G, et al. Comprehensive Molecular Portraits of Invasive Lobular Breast Cancer. Cell. 2015;163:506–19. 26451490 10.1016/j.cell.2015.09.033 PMC4603750

[103] Rosenbluth JM, et al. Organoid Cultures From Normal and Cancer-prone Human Breast Tissues Preserve Complex Epithelial Lineages. Nat Commun. 2020. 10.1038/s41467-020-15548-7. 32249764 10.1038/s41467-020-15548-7 PMC7136203

[104] Sachs N, et al. A Living Biobank of Breast Cancer Organoids Captures Disease Heterogeneity. Cell. 2018. 10.1016/j.cell.2017.11.010. 29224780 10.1016/j.cell.2017.11.010

[105] Chan J, et al. Lineage Plasticity in Prostate Cancer Depends on FGFR and JAK/STAT Inflammatory Signaling. Eur J Cancer. 2022;174:S4. 10.1126/science.abn0478 PMC9653178 35981096

[106] Karthaus WR, et al. Regenerative Potential of Prostate Luminal Cells Revealed by Single-cell Analysis. Science. 2020;368:497. 32355025 10.1126/science.aay0267 PMC7313621

